# Mental health-related, existential, and biological factors are associated with the desire to hasten death in Mexican cancer patients undergoing palliative care: A single-center study

**DOI:** 10.1371/journal.pone.0329736

**Published:** 2025-08-07

**Authors:** Oscar Rodríguez-Mayoral, Edith Monreal-Carrillo, Irazú Contreras-Yáñez, Silvia Allende-Pérez, Evandro Agazzi, Virginia Pascual-Ramos

**Affiliations:** 1 Mental Health Department, Instituto Nacional de Cancerología, Mexico City, Mexico; 2 Palliative Care Service, Instituto Nacional de Cancerología, Mexico City, Mexico; 3 Immunology and Rheumatology Department, Instituto Nacional de Ciencias Médicas y Nutrición “Salvador Zubirán” (INCMyN SZ), Mexico City, Mexico; 4 Inderdisciplinary bioethics center, Faculty of Health Sciences, Universidad Panamericana, Mexico City, Mexico; Instituto Mexicano del Seguro Social, MEXICO

## Abstract

**Introduction:**

The wish to hasten death (WTHD) is a clinically significant phenomenon that arises from complex suffering. It has been predominantly studied in Caucasian populations, emphasizing the importance of gaining more diverse cultural perspectives. This study explores the factors associated with the WTHD in Mexican cancer patients receiving palliative care from one academic center, with a specific focus on its connection to dignity.“.

**Patients and methods:**

The study, a cross-sectional research conducted between October 12, 2023, and August 30, 2024, involved patients with confirmed cancer diagnoses who were attending a palliative care service. Patients had applied the Patient Dignity Inventory (PDI), Schedule of Attitudes Toward Hastened Death (SAHD), Brief Edinburgh Depression Scale (BEDS), EORTC QLQ-C15-PAL to assess health-related quality of life, Karnofsky Performance Status Scale (KPSS) to assess functional capacity, and the Edmonton Symptom Assessment System. A PDI score ≥55 indicated a fractured sense of dignity (DPD), while a SAHD score ≥1 indicated the WTHD. Factors associated with the WTHD were identified using multiple logistic regression analysis. The study was approved by the IRB.

**Results:**

The study included 302 primarily middle-aged (54.5 [45–64]) females (225 [74.5%]), with 9 years of education. They reported high severity of well-being (7 [1–7]) and tiredness (3 [0–6]). Their median KPSS score showed independence (80 [70–80]), despite impacts across all EORTC QLQ-C15-PAL dimensions. DPD was noted in 110 patients (36.5%). The most frequent diagnoses were breast cancer (114 [38%]), lung cancer (33 [11%]), and gastrointestinal cancer (28 [9%]).

The WTHD was found in 94 patients (31.1%). Factors significantly associated included tiredness score (OR: 1.147, 95% CI: 1.044–1.261, p = 0.004), BEDS score (1.181, 1.085–1.284, p ≤ 0.001) and a DPD (1.979, 1.038–3.772, p = 0.04).

**Conclusions:**

The WTHD was found in one out of every three Mexican cancer patients receiving palliative care and was linked to biological-, mental health-, and existential-related factors.

## Introduction

The wish to hasten death (WTHD) is a complex, ambivalent, and fluctuating phenomenon that has been widely studied in the context of life-limiting conditions [1–6]. In a recent systematic overview, Rodríguez-Prat et al. [1] propose that the desire for death may be considered as existing along a continuum, defined by the extent to which thoughts of dying are linked to action. To improve understanding of the WTHD concept, an international Delphi process involving experts from Europe, USA and Canada developed an operational definition from and within the palliative care context [7]. According to that definition, the WTHD is a reaction to suffering in the context of a life-threatening condition, from which the patient can see no way out other than to accelerate his or her death. This wish may be expressed spontaneously or after being asked about it, but it must be distinguished from the acceptance of impending death or from a wish to die naturally, although preferably soon.

There is a lot of variation in the prevalence rates of WTHD across different studies. Two systematic reviews found rates ranging from 1.5% to 56%. These rates depend on the specific questionnaires applied, the questions asked and the score thresholds used [3,5]. In 2017, Bellido-Pérez et al. [5] analyzed 50 primary studies and identified seven different instruments used to measure WTHD, with the Schedule of Attitudes toward Hastened Death (SAHD) being one of the most commonly used. Interestingly, a short form of the SAHD has been developed, with versions adapted for USA [8], Spanish [9], and Mexican [10] populations. The validity of these versions is consistent with that of the original instrument [1]. In the most recent systematic overview, Rodríguez-Prat et al. [1] identified two additional instruments [11,12], which application has been extended to patients with amyotrophic lateral sclerosis [9].

Many factors have been associated with, serve as mediators of, or are predictive of the WTHD. These factors have the same multidimensional origin as the phenomenon of the WTHD itself, encompassing physical [1,13,14], psychological/emotional [1,14–18], existential/spiritual [2,14,19–25], and social domains [1,26]. Depression has been one of the most commonly reported emotional factor, emphasizing the importance of addressing it effectively [1]. A recent systematic review by Quah et al. [24] highlights that a loss of, or the fear of a loss of, dignity or autonomy are among the more prominent sources resulting in a patient’s WTHD and their request for assisted dying. The authors proposed that alleviating these fears reduced the WTHD and highlighted Gentzler’s argument that a similar outcome can be accomplished simply by ensuring patients have the legal right to request assisted dying [27]. Importantly, this review [1,24] identifies largely Western concepts of dignity within the assisted dying debate, and the majority of the publications were from the USA, UK, and the Netherlands.

The WTHD topic needs to be addressed by taking into account the cultural and anthropological aspects of the phenomenon. Rodríguez-Prat and van Leeuwen [6] analyzed the statements made by patients expressing a WTHD in relation to different classes of assumptions and identified four classes: Assumptions related to dignity, autonomy, and authenticity; social interactions; the value of life; and medicalization. These are greatly influenced by cultural background [27,28] and access to healthcare, which has particular characteristics in developed countries with fragile healthcare systems and pervasive inequities [29–31].

In particular, a” paternalistic” ideal of autonomy has been described as a frequent attitude among Mexican rheumatologists particularly in the context of public health system and Mexican patients with rheumatic diseases [32]. Thompson et al [33] have also suggested that paternalism, may be a more highly valued ethic in some cultural contexts, such as the Latino patients from USA, in whom the sole emphasis on patient autonomy could potentially have negative consequences such as the alienation of Latino patients and lower rates of treatment adherence.

From a human rights perspective, dignity is viewed as a universal need essential for the well-being of every individual in all societies. It is inherently linked to human rationality and morality and is acknowledged as not dependent on or varying with circumstances [34–36]. This understanding of dignity is recognized as intrinsic, basic, or human dignity [34]. Sulmasy has suggested that any other interpretation of “dignity” should be logically and linguistically subordinate to the concept of intrinsic dignity [34]. However, in healthcare settings, the notion of dignity is connected to patients’ values. It reflects how patients perceive themselves and how they are perceived by others, as well as how the nature of their illness affects their life and identity [37]. In this context, dignity is generally equated with a person’s sense of autonomy and control [37–40]. This notion of dignity is recognized as social, attributed or perceived dignity [34,37]. Most empirical work looking at dignity in health pertains to the social notion of dignity [37].

To date, most current knowledge on the of the WTHD is based on clinical evidence obtained from primarily Caucasian populations, with underrepresentation of patients from the Latin American (LATAM) region, which limits its comprehensiveness.

The objective of the study was to identify factors associated with WTHD in cancer patients receiving palliative care at a single academic center in Mexico City. We hypothesized a potential association between perceived dignity and the WTHD and the hypothesis was confirmed.

## Ethical considerations

The Research Ethics Committee of the Instituto Nacional de Cancerología (INCan) approved the study (Reference number: CEI/018/23 and 023/064/CPI). All the patients included in the study provided written informed consent.

The informed consent process in Mexico typically includes several key components to ensure participants are fully informed. First, participants were informed about the purpose of the research, including its objectives and anticipated benefits. A brief description of the questionnaires used and the duration of the study for each participant and the whole study was also provided. Additionally, participants received an explanation of any potential discomfort associated with participation along with the expected benefits. Confidentiality was emphasized, assuring participants that their personal data and results will be kept confidential and that their identity will not be disclosed without their consent. It was also made clear that participation was voluntary; participants could withdraw at any time without penalty or loss of benefits from the institution conducting the study. To address any questions or concerns, participants were given time and also contact information for investigators and ethics boards. Lastly, it was ensured that participants fully understood the information provided and had the opportunity to ask questions before giving their consent. The entire informed consent process was documented for record-keeping purposes.

## Patients and methods

### Setting and study population

The INCan is a tertiary care level and national referral center for cancer patients, located in Mexico City. The study was conducted from October 12, 2023, to August 30, 2024. During the study period 2613 patients had follow-up scheduled consultations at the palliative care service (PCS) (Departamento de Bioestadística INCan, 2024 data). The most common primary sites for cancer at the PCS are listed in Table 1.

**Table 1:** Prevalence of primary sites for cancer at the PCS, during the study period.

	Patients referred for follow-up evaluations at the PCS, n = 2613
Gastrointestinal cancer and liver and bile duct cancer	617 (23.6)
Breast cancer	487 (18.6)
Urologic cancer	393 (15)
Gynecologic cancer	370 (14.2)
Skin and soft tissue cancer	222 (8.5)
Thorax cancer	184 (7)
Hematologic cancer	176 (6.7)
Head and neck cancer	154 (5.9)
Unknown	10 (0.4)

*Data are presented as number (%) of patients.*

Consecutive outpatients who had received a biopsy-proven cancer diagnosis and had a scheduled follow-up evaluation at the PCS were invited to participate (inclusion criteria). Patients with uncontrolled physical symptoms, delirium, psychotic symptoms, or severe cognitive impairment, as determined by the attending psychiatrist assigned to the PCS, were excluded from the study.

### Study design and patients’ assessments

**Fig 1:**
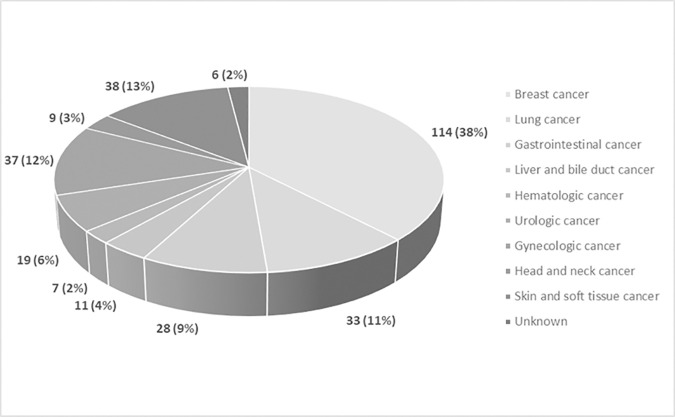
Primary cancer diagnoses distribution. Data are presented as number (%) of patients.

The study was cross-sectional. Potential participants were identified at the PCS while waiting for a scheduled consultation. A co-investigator briefly explained the study’s aim and asked if the patients were interested in participating. Upon agreement from the patients, the informed consent process was carried out. The following Spanish/Mexican validated versions of the questionnaires (but for the Karnofsky Performance Status Scale [KPSS]) were then administered to the patients in the specified order: The Patient Dignity Inventory (PDI) [41], the Schedule of Attitudes Toward Hastened Death (SAHD) [10], the Brief Edinburgh Depression Scale (BEDS) [42], the European Organization for Research and Treatment of Cancer quality of life questionnaire (EORTC QLQ-C15-PAL) [43], the KPSS for functional capacity [44], and the Edmonton Symptom Assessment System (ESAS-S) [45]. The questionnaire applications were self-administered, but assistance was available when needed. Patients spent an hour completing the questionnaires. In addition, investigators used standardized formats to collect sociodemographic variables, including Adolescents and Young adults (AYAs) status [46] (Table 2), other cancer-related variables (Table 3) including specific cancer diagnosis (Fig 1), and mental health-related variables (Table 4). The data was verified through careful chart review and patient interviews.

**Table 2:** Description of the population´s sociodemographic characteristics and the comparison between patients with and without the WTHD.

	Overall population n = 302	Patients without the WTHD, n = 208	Patients with the WTHD, n = 94	p value
Age, years	54.5 (45-64)	55 (45-65)	54 (44.8-61.3)	0.170
Years of age at cancer diagnosis	50 (41-61)	51 (41-61)	49 (38.8-60)	0.261
Females*	225 (74.5)	160 (76.9)	65 (69.1)	0.157
AYAs status*	50 (16.6)	32 (15.4)	18 (19.1)	0.409
Years of scholarship	9 (6-12)	9 (6-12)	9 (6-12.5)	0.236
Marital status*				
* Single/Divorced/Widow*	133 (44)	48 (43.1)	35 (37.2)	0.07
* Living together*	169 (56)	110 (52.9)	59 (62.8)	
Occupation*				
* Formal and non-formal job*	118 (39.1)	77 (37)	41 (43.6)	0.249
* Unemployed*	60 (19.4)	39 (18.8)	21 (22.3)	
* Household wife*	124 (41.1)	92 (44.2)	32 (34)	
Self-referred with religious beliefs*	297 (98.3)	203 (97.6)	94 (100)	0.249
Time from home to hospital transportation (hours)	2.5 (1.5-3.5)	2 (1.5-3.5)	2.5 (1.5-3.5)	0.294
Modality of home to hospital transportation*				
* Public*	171 (59)	122 (60.4)	49 (55.7)	0.754
* Private*	107 (36.9)	72 (35.6)	35 (39.8)	
* Ambulance*	12 (4.1)	8 (4)	4 (4.5)	

Data presented as median (IQR) or otherwise indicated. *Number (%) of patients.

**Table 3:** Description of the population´s cancer-related characteristics and the comparison between patients with and without the WTHD.

	Overall population n = 302	Patients without the WTHD, n = 208	Patients with the WTHD, n = 94	p value
ESAS scores				
* Pain*	2 (0-5)	2 (0-4)	3 (0-6)	0.010
* Tiredness/fatigue*	3 (0-6)	2 (0-5)	5 (2-7)	≤0.0001
* Nausea*	0 (0-2)	0 (0-2)	0 (0-2.3)	0.584
* Depression*	2 (0-6)	0 (0-4)	5 (0-7)	≤0.0001
* Anxiety*	0 (0-4)	0 (0-2)	3 (0-6)	≤0.0001
* Drowsiness*	2 (0-5)	1.5 (0-4)	2.5 (0-6)	≤0.0001
* Loss of Appetite*	0 (0-5)	0 (0-4)	3 (0-5.3)	0.072
* Wellbeing*	5 (1-7)	5 (0-8)	5 (3-7)	0.092
* Shortness of breath*	0 (0−0)	0 (0−0)	0 (0-3)	0.016
* Sleep quality*	0 (0-4)	0 (0-3)	2 (0-6)	0.004
* Spiritual pain*	0 (0-5)	0 (0-3)	3 (0-7)	≤0.0001
* Economic concern*	0 (0-3)	2 (0-5)	5 (0-7)	0.003
KPSS score	80 (70-80)	80 (70-90)	70 (60-80)	0.002
EORTC QLQ-C15-PAL				
*Symptoms scale score*	70.4 (51.9-85.2)	77.8 (63-88.8)	59.3 (48.2-74.1)	≤0.0001
*Physical function scale score*	77.8 (55.4-100)	88.9 (66.7-100)	66.7 (44.4-89)	≤0.0001
*Emotional function scale score*	66.7 (50-100)	83.3 (66.7-100)	66.7 (33.3-70.9)	≤0.0001
*Overall quality of life*	66.7 (50-83.3)	66.7 (50-83.3)	66.7 (33.3-66.7)	≤0.0001
PDI score	44.5 (35-62)	39.5 (32-54.8)	61 (43.8-77)	≤0.0001
Factor 1 score (Loss of meaning in life)	18 (14-27)	16 (13-22)	26 (18.8-32.3)	≤0.0001
Factor 2 score (Anxiety and loss of autonomy)	20 (14-26)	17 (13-22)	25 (18.8-31.3)	≤0.0001
Factor 3 score (Dependency)	2 (2-4)	2 (2-4)	3 (2-5.3)	0.001
Factor 4 score (Social support)	3 (3-5)	3 (3-4)	4 (3-6)	0.002
PDI score ≥55*	110 (36.4)	52 (25)	58 (61.7)	≤0.0001

Data presented as median (IQR) or otherwise indicated. *Number (%) of patients.

**Table 4:** Description of the population´s mental-health-related characteristics and the comparison between patients with and without the WTHD.

	Overall population n = 302	Patients without the WTHD, n = 208	Patients with the WTHD, n = 94	p value
Previous mental health comorbidity*				
*None*	256 (86.2)	180 (87)	76 (84.4)	
*Depression*	26 (8.8)	16 (7.7)	10 (11.1)	0.760
*Anxiety*	9 (3)	7 (3.4)	2 (2.2)	
*Other (five missing data)*	6 (2)	4 (1.9)	2 (2.2)	
Current tobacco use*	55 (18.3)	39 (18.9)	16 (17)	0.750
Current alcohol consumption*	81 (27)	55 (26.7)	26 (27.7)	0.889
BEDS score	7 (3-10)	5 (2-8)	10 (6-12)	≤0.0001
Depression according to BEDS cut-off*	97 (32.1)	42 (20.2)	55 (58.5)	≤0.0001
Suicidal behavior (item 6 from BEDS)*	66 (21.9)	18 (8.7)	48 (51.1)	≤0.0001

Data presented as median (IQR) as otherwise indicated. *Number (%) of patients.

### Questionnaires´ description

#### The PDI.

This is a validated version of the original PDI for Mexican cancer patients [41]. The original PDI was developed for Canadian patients with cancer [47]. The inventory consists of 25 items grouped into four factors. Each item is rated on a five-point Likert scale, where 1 means “not a problem” and 5 means “an overwhelming problem.” The final PDI score ranges from 25 to 125, with higher scores indicating a greater loss of dignity.

#### The SADH.

The (short version) questionnaire consists of six items grouped into one factor and has been widely used to assess the WTHD among patients with life-threatening conditions. Each item is rated as false (0 points) or true (1 point). The final score ranges from 0 to 6, with higher scores indicating a greater WTHD [10].

#### The BEDS.

The BEDS was constructed from the Edinburgh Postnatal Depression Scale, a 10-item self-rating scale, as a case-finding tool for depression specifically in patients with advanced cancer [42]. The shortened version includes six items, each rated on a 4-point scale. Item 6 evaluates suicidal behavior. The global score ranges from 0 to 18, with higher scores indicating more severe symptoms of depression. The authors suggest a BEDS score of ≥9 for diagnosing depression, with a sensitivity of 28% and specificity of 92.9% [42].

#### The EORTC QLQ-C15-PAL.

The questionnaire is a shortened 15-item version of the QLQ-C30 [48], which was designed to assess the health-related quality of life in patients with advanced, incurable, and symptomatic cancer. This version focuses on key aspects to patients such as physical and emotional function, pain, fatigue, nausea/vomiting, appetite, dyspnea, constipation, sleeping difficulties, and overall quality of life. Each item is rated on a four-point Likert scale, with 1 indicating “not at all” and 5 indicating “very much.” The questionnaire includes four dimensions: symptoms scale, physical function scale, emotional function scale, and overall quality of life. The final score, after a linear transformation, ranges from 0 to 100, with higher scores representing better health-related quality of life.

#### KPSS.

The KPSS scale was the first tool developed to assess functional capacity in cancer patients. It measures various functional aspects, including activity level, work capacity, level of care needed, disease severity and progression, and the need for hospitalization. This scale ranges from 0 (indicating death) to 100 (indicating normal health) and assigns higher scores to higher function. The scores have prognostic value, meaning that in most serious illnesses, a lower score indicates a worse likelihood of survival. A KPSS score of 70 is the threshold for defining totally autonomous individuals [44].

#### ESAS.

This scale is used to assess the main physical and psychological symptoms in patients receiving care from palliative care teams. It uses a numerical rating scale to evaluate 12 symptoms: pain, fatigue/tiredness, nausea, sadness, anxiety, drowsiness, poor appetite, well-being, shortness of breath, insomnia, spiritual pain, and financial worries. The patient is asked to rate the severity of each symptom on a scale from 0 to 10, with higher scores indicating more symptoms severity [45].

### Definitions

We established that a sense of fractured dignity was present when patients scored the PDI ≥ 55, based on the 75th percentile of the data distribution (personal communication) [41].

We defined the WTHD as a SADH questionnaire score of 1 or higher [10].

### Sample size calculation

In a previous study, researchers found significant differences in PDI scores between patients with and without the WTHD [49]. They observed a difference of 10.5 points between the scores. To estimate the sample size, we hypothesized a medium effect size of Cohen’s d = 0.4 in a non-normal distribution, and considered a two-tailed test, 5% significance level, and 80% power. We determined that at least 234 patients would be needed. Considering a 20% potential loss of non-analyzable data, the number was increased to (at least) 281 patients.

### Statistical analysis

We conducted a descriptive statistical analysis, showing the frequencies for categorical variables and the median (interquartile range, [IQR]) for numerical variables.

Spearman’s correlation coefficient was used to assess the strength and direction of the relationship between dignity and the WTHD.

We used multiple logistic regression analysis to identify factors associated with the WTHD (dependent variable). We created a global model where variables were included based on their statistical significance in the univariate analysis (p ≤ 0.0015 after Bonferroni correction for multiple comparisons). Test-based forward and backward selection were used to determine the final model. We examined correlations between variables to avoid overfitting the models, but none were relevant (rho value ≥0.70). The Nagelkerke pseudo-R^2^ test is reported as a measure of model fit goodness. Results are expressed as adjusted Odds Ratios (exponentiated regression coefficients, exp[β]) and their 95% confidence interval (95%CI).

The amount of missing data was below 1%, and no imputation was performed.

All statistical analyses were conducted using Statistical Package for the Social Sciences version 21.0 (SPSS Chicago IL). A p-value of <0.05 was considered statistically significant.

## Results

### Population characteristics

During the study period, we included 302 patients, whose characteristics and main diagnoses are summarized in Tables 2 and 4 and Fig 1.

Briefly, the patients were mostly middle-aged (54.5 [45–64]) females (225 [74.5%]) with an average of 9 years (6–12) of formal education. The majority of them lived together (169 [56%]), were household wife (124 [41.1%]), and had self-reported religious beliefs (297 [98.3%]). On average, patients spent 2.5 hours traveling from home to the hospital, primarily using public transportation services. Additionally, 50 patients (16.6%) had AYAs status (Table 2). According to the ESAS, patients scored higher (indicating more severity) in the following symptoms: well-being (7 [1–7]), tiredness/fatigue (3 [0–6]), depression (2 [0–6]), pain, and drowsiness (2 [0–5]). The median KPSS score indicated that patients were independent (80 [70–80]), although all dimensions of their health-related quality of life were affected. A significant number of patients (110 [36.5%]) reported a fractured sense of dignity (Table 3). Finally, the majority lacked previous mental health comorbidity although 97 (31.1%) and 66 (21.9%) had depression and suicidal behavior, respectively, based on BEDS cut-offs (Table 4).

Most common diagnoses were breast cancer (114 [38%]), lung cancer (33 [11%]), and gastrointestinal cancer (28 [9%]) (Fig 1).

### The WTHD phenomenon and its association with perceived dignity

There were 94 patients (31.1%) who scored ≥1 on the SADH and had WTHD. The PDI and SADH showed a mild but significant correlation, rho = 0.41, p ≤ 0.001.

### Comparison of patient characteristics between those who scored the WTHD and their counterparts

The results in Tables 3 and 4 show that patients with the WTHD scored higher on ESAS symptoms severity, except for nausea, loss of appetite, and wellbeing. They also scored higher on the KPSS, the four dimensions of the EORT-QLQ-C15-PAL, the global PDI score, each of the four PDI factor scores, and the BEDS score compared to patients without the WTHD. Additionally, more patients with the WTHD reported a fractured sense of dignity, as well as depression and suicidal behavior based on the BEDS cut-offs.

### Factors associated with the WTHD

**Fig 2:**
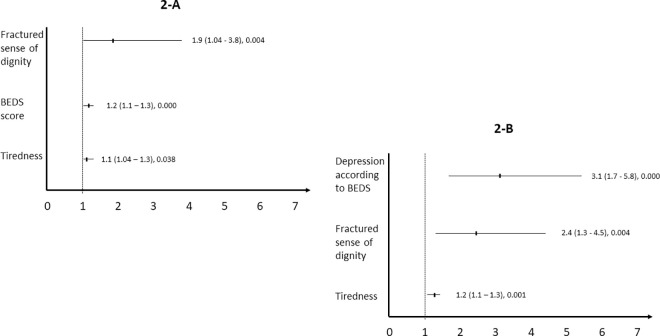
Factors associated with the WTHD. The figure displays the odds ratio, 95% confidence interval, and p-value for each variable significantly associated with the WTHD. The BEDS contributes to both models as a continuous variable (2-A) and as a dichotomous variable (2-B).

Different models were tested and they all showed similar results. Fig 2A summarizes the results from the model that considered the following variables and test-based forward selection: tiredness/fatigue, depression, anxiety, drowsiness, spiritual pain, the four domain scores of the EORTC-QLQ-C15-PAL, a fractured sense of dignity (PDI score ≥55), and the BEDS score. Tiredness/fatigue score, BEDS score, and a fractured sense of dignity were the variables significantly associated with the WTHD (Nagelkerke Pseudo R² = 0.282). The model was repeated, replacing the BEDS score with depression according to BEDS cut-off, and the results were similar, with variables showing even a stronger association with the WTHD, as shown in Fig 2B (Nagelkerke Pseudo R² = 0.271). Finally, the optimal cut-off for tiredness/fatigue score to predict the WTHD was 4.5 (sensitivity: 0.596, specificity: 0.722, Area Under the Curve: 0.670).

## Discussion

The study aimed to identify the factors related to the WTHD in a specific group of Mexican cancer patients receiving palliative care. It focused on perceived dignity and used validated questionnaires to assess both dignity and the WTHD. The WTHD is a complex concept, and its measurement should involve conceptually defining the variable and developing an appropriate instrument to quantify it accurately in order to avoid bias. The study assessed the phenomenon with an adapted and validated Mexican short version of the original SADH in cancer patients [50]. A systematic review of different instruments used to measure the WTHD found that while the SADH has limitations, it remains the most widely used instrument to date, allowing for comparisons of results, and has been most frequently analyzed for its psychometric properties. Additionally, the SADH is available in multiple languages and is designed more for research purposes [5].

First, we found that one in three Mexican cancer patients receiving palliative care experienced the WTHD. Our prevalence rate is on the higher end of the range of rates described in systematic reviews, which have highlighted considerable variability in prevalence rates [1,2,4,5]. This variability could be due to the lack of precision in the terminology used to describe the WTHD (e.g., thoughts of dying, genuine wish to die, wish to die, desire to hasten death, desire for early death, etc...) [1], the use of different standardized assessment instruments and varying instrument cut-off scores to define the WTHD [5], the voluntary expression of WTHD without any prompting, encouragement, or being directly asked by the assessor during the care planning process [51], and anthropological factors influencing how patients experience and give meaning to the WTHD [2,4]. In a review of assessment instruments, Bellido-Perez et al. [5] observed that the proportion of patients manifesting the WTHD ranged from 1.5% to 35%, depending on the specific instrument and cut-off score used. Two Spanish studies reported a much higher WTHD prevalence when assessed with the Assessment of the Frequency and Extent of Desire to Die questionnaire (18%) compared to the SADH-5 (8.8%) [51,52]. Our high prevalence may be linked to unique anthropological factors in our country, especially social aspects that pose challenges to healthcare and the healthcare system [30]. In a previous study involving a similar population, it was found that 44% of the 64 Mexican cancer patients receiving palliative care and undergoing a psychiatric evaluation had WTHD. WTHD was defined as the presence of thoughts of death, suicidal ideation, and/or a request for euthanasia or medical assistance in dying [53].

Second, we found three variables/factors linked to the WTHD, each corresponding to a different aspect of human experience. Tiredness/fatigue score relates to the biological domain, the BEDS score to the mental health domain, and a fractured sense of dignity to the existential domain. These factors were significantly associated with the WTHD. In the model, a fractured sense of dignity had the most pronounced effect. However, when we replaced the BEDS score with its most severe expression, namely the diagnosis of depression, this variable had the strongest impact.

Literature reviews have consistently found that socio-demographic factors, such as younger age, higher socioeconomic status, and lower religiosity are associated with or predictive of the WTHD [1,3,54,55]. However, we were unable to identify a specific sociodemographic variable associated with the phenomenon in our population, possibly due to how the construct was defined, the variables distribution in our population (religious beliefs were referred by 98% of the patients) and more important independent effect of psychological and existential symptoms, particularly depression, in the WTHD.

Consistent with our findings, depression has been linked with the WTHD [1], and in some studies, it has shown the strongest association with this phenomenon [56]. This connection has been supported by studies utilizing various methodologies. Some studies have found a higher prevalence of depression among WTHD patients [12,57], as well as positive correlations between different assessment tools [18]. Other studies have employed more robust methods, such as regression analysis [49,58] and factorial analysis [51] to confirm depression as predictors to the WTHD. Additionally, depression has been identified as a strong mediator in models of the WTHD [20,59,60]. It is important to acknowledge the benefits of the BEDS for assessing depression in cancer patients. First, the BEDS is designed to capture the nuanced symptoms of depression, which can often be complicated by the physical and emotional challenges faced by cancer patients, making it particularly useful for this population. Second, the scale is user-friendly and can be completed quickly, allowing healthcare providers to integrate it into routine assessments without burdening patients [42]. Third, the BEDS has strong psychometric properties, including both validity and reliability, which means it consistently measures what it intends to—the presence and severity of depressive symptoms—making it a trustworthy choice for clinicians [42]. Finally, depression in cancer patients can manifest differently than in other populations, and the BEDS takes into account the unique challenges and life stressors that these patients face, offering a more tailored approach to screening.

In studies involving terminally ill patients, three literature reviews have supported our findings and have shown that requests for assisted dying (a narrow approximation to the WTHD) are often driven by fears or the presence of a loss of dignity among patients and their families [2,4,61]. Quah et al. [24] conducted a systematic scoping review to examine the current arguments in the assisted dying debate through the lens of the ring theory of personhood. This theory suggests that concepts of personhood can be represented by the innate, individual, relational, and societal rings, each containing specific values, beliefs, and principles that inform their corresponding identities. Together, these rings embody a particular notion of dignity and are sensitive and adaptable to detect and map changes in concepts of personhood [62]. The study concludes that concepts of dignity constantly evolve and fluctuate throughout a patient’s end-of-life journey and are a proxy for sustaining self-concepts of personhood and how patients wish to be perceived by others. The fear of losing dignity or autonomy, which are considered synonymous in healthcare literature, is a significant factor leading to requests for assisted dying [62]. It’s important to note that this connection may be influenced by aging, which could potentially protect against the feeling of losing dignity [63], although the research findings have not been consistent [64].

Several articles have confirmed that the severity of symptoms experienced by terminally ill patients increases their level of WTHD [22,23,49,65]. This might be particularly relevant with symptoms that interfere with mood and relations with other people [66]. This relationship has been described in terms of pain or symptom burden as a whole [22], as well as specific symptoms such as dry mouth [67], drowsiness [25,67], incontinence [51], or, as observed in our study, tiredness [25,49,67]. It is interesting to note that in our study, the optimal cut-off for tiredness/fatigue score to predict the WTHD was 4.5. The cutoff point appears to be low considering the 0–10 scale, where 0 indicates the symptom is not present and 10 indicates the most severe state. This highlights the significance of optimizing symptom management in patients who are receiving palliative care. However, we also observed that tiredness score was much less significant in the model than psychological and existential variables. Kelly et al. [51] have confirmed our results regarding physical symptoms and suggested that the distress patients associate with them may represent a separate dimension to psychological symptoms such as depression. This might challenge the idea of assisted suicide or euthanasia as a justifiable and appropriate response to “incurable physical suffering.”

The study results highlight the need for some ethical considerations. The concept of the WTHD is currently seen as a reflection of human suffering, with similar reasons observed across different patient populations [1,2]. Many of these factors can be addressed or reduced through medical interventions (for biological and emotional aspects) and interventions focused on preserving dignity and finding meaning in life (for existential factors), both of which are integral to clinical ethics. Qualitative studies of the WTHD reveal that such a wish can have different meanings, all of which are deeply connected to the patient’s personal and sociocultural background. These meanings do not necessarily imply a desire to end one’s life or request euthanasia/assisted suicide [1,2]. Additionally, there is evidence that the phenomenon changes over time among terminally ill cancer patients [68] and can be influenced by the specific nature of the different dying trajectories (neurological disease, organ failure, frailty due to age and cancer), which impact agency and self-understanding [26].

According to the bioethical framework of the “four-principles approach” (which is based on the principles of autonomy, beneficence, non-maleficence, and justice) [20], there might be a dilemma when patients express the WTHD. This dilemma arises because physicians must decide whether to prioritize the patient’s autonomy or the principle of beneficence [69]. In the case of patients with a concurrent mental disorder, the expression of WTHD may be seen as a cry for help rather than a true desire to die [70]. This leads to a conflict between respecting the patient’s right to self-determination and the duty to act in the patient’s best interest, which might be to prioritize interventions directed to the underlying mental disorder.

The bioethicist Diego Gracia [71] argues that autonomy and beneficence are linked to an individual’s belief system and personal life goals, while justice and non-maleficence are related to ensuring survival and setting a minimum standard for decision-making. This framework suggests that autonomy cannot be upheld if non-maleficence is compromised, for example, in cases of euthanasia or assisted dying.

Therefore, we propose that when a patient expresses a WTHD, the first step should be to address their physical, emotional, and existential symptoms, interpersonal concerns, and overall suffering before considering any further action.

### Limitations

The study has several limitations that need to be addressed. First, it was conducted at a single institution, and the sample collection was from the PCS, which may have biased the results. Second, the cross-sectional design of the study prevents the establishment of causal relationships between the variables. Third, there was heterogeneity in cancer diagnoses among the participants, although all of them had advanced stage disease. Additionally, other relevant diagnoses in the local epidemiology, such as gastrointestinal cancer, were not represented. Fourth, participants with severe psychiatric disorders were excluded from the study, which may have led to a bias in favor of patients with lower levels of the WTHD. Fifth, depression was diagnosed based on the BEBS score instead of through a psychiatric evaluation as recommended [72]. Finally, we considered a limited number of variables as potential factors associated with the WTHD.

## Conclusions

In conclusion, our study revealed that one in three Mexican cancer patients receiving palliative care experienced WTHD in a single center located in Mexico City. We identified three factors associated with WTHD, each corresponding to different aspects of the human experience: the tiredness/fatigue score (related to the biological domain), the BEDS score (linked to mental health), and a diminished sense of dignity (connected to the existential domain). Notably, the diminished sense of dignity had the most significant effect, with an exponentiated regression coefficient of 1.9. However, when we substituted the BEDS score with its most severe clinical expression—diagnosis of depression—this variable showed the greatest impact, with the exponentiated regression coefficient increasing to 3.1. It is interesting to note that these factors share the same multidimensional origin as WTHD itself, reinforcing the idea that WTHD is a clinically significant phenomenon primarily arising from multifactorial suffering.
